# Mapping assays to the key characteristics of carcinogens to support decision-making

**DOI:** 10.1093/database/baaf026

**Published:** 2025-04-22

**Authors:** Gabrielle Rigutto, Cliona M McHale, Ettayapuram Ramaprasad Azhagiya Singam, Iemaan Rana, Luoping Zhang, Martyn T Smith

**Affiliations:** Division of Environmental Health Sciences, School of Public Health, University of California, 2121 Berkeley Way, Berkeley, CA 94704, United States; Division of Environmental Health Sciences, School of Public Health, University of California, 2121 Berkeley Way, Berkeley, CA 94704, United States; Molecular Graphics and Computation Facility, College of Chemistry, University of California, 175 Tan Hall, Berkeley, CA 94720, United States; Division of Environmental Health Sciences, School of Public Health, University of California, 2121 Berkeley Way, Berkeley, CA 94704, United States; Division of Environmental Health Sciences, School of Public Health, University of California, 2121 Berkeley Way, Berkeley, CA 94704, United States; Division of Environmental Health Sciences, School of Public Health, University of California, 2121 Berkeley Way, Berkeley, CA 94704, United States

## Abstract

The key characteristics (KCs) of carcinogens are the properties common to known human carcinogens that can be used to search for, organize, and evaluate mechanistic data in support of hazard identification. A limiting factor in this approach is that relevant *in vitro* and *in vivo* assays, as well as corresponding biomarkers and endpoints, have been only partially documented for each of the 10 KCs (Smith MT, Guyton KZ, Kleinstreuer N *et al*. The key characteristics of carcinogens: relationship to the hallmarks of cancer, relevant biomarkers, and assays to measure them. *Cancer Epidemiol Biomarkers Prev* 2020;**29**:1887–903. https://doi.org/10.1158/1055-9965.EPI-19-1346). To address this limitation, a comprehensive database is described that catalogues these previously described methods and endpoints/biomarkers pertinent to the 10 KCs of carcinogens as well as those referenced as supporting evidence for each KC in the International Agency of Research on Cancer Monograph Volumes 112–131. Our comprehensive mapping of KCs to assays and endpoints can be used to facilitate mechanistic data searches, presents a useful tool for searching for assays and endpoints relevant to the 10 KCs, and can be used to create a roadmap for utilizing data to evaluate the strength of the evidence for each KC. The KC-Assay database is available to the public on the web at https://kcad.cchem.berkeley.edu and acts as a ‘living document’, with the ability to be updated and refined.

**Database URL**: https://kcad.cchem.berkeley.edu

## Introduction

### Mechanistic evidence in the cancer hazard assessment process

The integration of mechanistic evidence into carcinogenic hazard assessments has been a foundational aspect of the International Agency for Research on Cancer (IARC) Monographs from their inception [1]. Historically, this evidence was principally employed to determine if an adjustment of the carcinogenicity classification derived from human and animal cancer data was warranted. With the 2019 update to the Monographs Preamble, the status of mechanistic evidence has been elevated. It is now considered in conjunction with evidence from cancer studies in humans and animals, acknowledging the growing quantity, variety, and significance of mechanistic data in recognizing cancer risks [2].

In a 2012 workshop convened by the IARC Monographs Programme, working group members evaluated existing information on the mechanisms of action for established human carcinogens (Group 1) and identified 10 key characteristics (KCs) of carcinogenic agents [3]. These include the following: (i) is electrophilic or capable of metabolic activation; (ii) is genotoxic; (iii) altering DNA repair/causing genome instability; (iv) induces epigenetic alterations; (v) induces oxidative stress; (vi) induces chronic inflammation; (vii) is immunosuppressive; (viii) modulating receptor-mediated effects; (ix) causes immortalization; and (x) alters cell proliferation, death or nutrient supply [4]. A more detailed summary of each of the 10 KCs is provided in Table 1. The KC approach [4, 5] has been systematically employed in analysing the mechanistic literature across 23 volumes of IARC Monographs, beginning in 2015 (see the Systematic extraction of the information from IARC Monographs section, Supplementary Table 1). For each monograph, the convened Working Group rigorously examines scientific data, sourced through targeted searches for each KC, including human epidemiological studies and experimental research, both *in vivo* and *in vitro* (also encompassing human primary cells and tissues in *ex vivo* applications) [2]. The accumulated expertise from evaluating these chemical agents/substances reveals that the KC approach has substantially refined the evaluation process of mechanistic evidence. Implementing the KC approach has not only standardized assessments but has also systematized the review of accessible mechanistic literature on carcinogens.

**Table 1.** The 10 KCs of carcinogens and relevant evidence

KC		Relevant evidence^a^
1	Is electrophilic or capable of metabolic activation	Parent compound or metabolite with an electrophilic structure (e.g. epoxide, quinone, etc.), formation of DNA and protein adducts
2	Is genotoxic	DNA damage (DNA strand breaks, DNA–protein cross-links, unscheduled DNA synthesis), intercalation, gene mutations, cytogenetic changes (e.g. chromosome aberrations, micronuclei)
3	Alters DNA repair or causing genomic instability	Alterations of DNA replication or repair (e.g. topoisomerase II, base excision, or double-strand break repair)
4	Induces epigenetic alterations	DNA methylation, histone modification, microRNAs
5	Induces oxidative stress	Oxygen radicals, oxidative stress, oxidative damage to macromolecules (e.g. DNA, lipids)
6	Induces chronic inflammation	Elevated white blood cells, myeloperoxidase activity, altered cytokine and/or chemokine production
7	Is immunosuppressive	Decreased immunosurveillance, immune system dysfunction
8	Modulates receptor-mediated effects	Receptor in/activation (e.g. ER, PPAR, and AhR) or modulation of endogenous ligands (including hormones)
9	Causes immortalization	Inhibition of senescence, cell transformation
10	Alters cell proliferation, cell death, or nutrient supply	Increased proliferation, decreased apoptosis, changes in growth factors, energetics and signalling pathways related to cellular replication or cell cycle control, angiogenesis

Abbreviations: AhR, aryl hydrocarbon receptor; PPAR, peroxisome proliferator-activated receptor.

a Based on description from the study by Smith *et al*. (2016).

### Project objective

The KCs of carcinogens were first described by Smith et al., 2016 [4]. They identify the properties common to IARC Group 1 human carcinogens. The KCs are now used by IARC, the US National Toxicology Program Report on Carcinogens, the US Environmental Protection Agency (EPA) Integrated Risk Information System programme, and California Environmental Protection Agency’s (CalEPA) Office of Environmental Health Hazard Assessment to inform decisions about the likelihood that a chemical is a carcinogen.

One of the current roadblocks to applying the KCs in decision-making is the lack of a systematic mapping of available assays and endpoints to each of the 10 KCs. A comprehensive mapping could facilitate and systematize searching for and organizing KC-relevant mechanistic data. Smith et al., 2020 [6] described a series of well-established and emerging assays that have been, or could be, used to measure each KC *in vitro/ex vivo* and *in vivo* in exposed humans or animals. This list was representative only and not intended to be fully comprehensive. The goal of the current project is to provide a more comprehensive mapping of published assays to each of the KCs, which may be used to generate a roadmap for using information from these assays and models in evaluating the strength of the evidence for each KC.

An additional motivation for this project is that for several KCs, there is a relative paucity of assays that are well-established as indicators of a chemical agent causing related KC endpoints. While the assays employed to measure the electrophilicity (KC1), genotoxicity (KC2), and capacity to induce oxidative stress (KC5) are relatively well-defined [7, 8], there has been regular debate within the scientific community over whether certain assays may serve as indicators of other KC endpoints, e.g. chronic inflammation (KC6). The IARC monographs provide a wealth of information, as the studies referenced therein have been systematically searched for and evaluated for relevancy. We recognize that the mechanistic evidence chapters have been historically used to identify endpoints that have been employed to substantiate evidence of a particular KC, and the references cited could provide specificity into the types of assays used/other pertinent information regarding study design or other experimental conditions.

## Methods

### Reference mining and extraction of the representative assay list(s) from the study by Smith et al., 2020 [6]

The primary publications cited in Tables 1 and 2 in the study by Smith et al., 2020 [6], which presented endpoints associated with each of the 10 KCs and the types of assays used to measure them, were reviewed in full to collect detailed information on the assays and their experimental designs (e.g. human *in vivo*, *in vitro*, and *ex vivo*). Other references cited in this publication, including many detailing emerging technologies used to measure the 10 KCs, were also reviewed. Review and data extraction processes are described further in the Systematic extraction of the information from IARC Monographs section.

### Systematic extraction of the information from IARC Monographs

IARC’s chemical classifications were downloaded from their website (https://monographs.iarc.who.int/list-of-classifications) to an Excel file. This list of agents was initially restricted to Monograph Volume 112 through Volume 131 (*N* = 36) and then further restricted to only ‘chemical’ agents (defined as those with a listed Chemical Abstracts Service Registry number, *N* = 34) and those with an IARC classification of 2A or 1 (*N* = 24). Full-text PDF files for each IARC Monograph from Volumes 112–131 were downloaded from the IARC Monographs website. A list of these chemical agents, their IARC classification, corresponding monograph number, and year of publication are presented in Supplementary Table 1.

For each of the 24 chemicals that satisfied the above conditions, the respective monograph was used to identify the KCs for which information was reported in the respective Chapter 4 (‘Mechanistic and Other Relevant Data’). As we have aimed to capture all potentially relevant endpoints and ensure as comprehensive of an assay list as possible, all KCs for which there was any evidence for each of these chemicals were recorded, regardless of the volume of evidence or the strength of the evidence provided.

KC-specific sections (accompanied by text headers) of the mechanistic data chapter in each monograph are typically organized according to experimental design and are presented in the following order: exposed humans, human primary cells, human cell lines, mammalian (*in vivo*, *ex vivo*, and *in vitro*) experimental systems, non-mammalian (*in vivo*, *ex vivo*, and *in vitro*) experimental systems, and *in silico/*modelling approaches as provided. References (first author name and year of publication) from each KC-specific section/experimental design subsection were extracted into their own distinct line item within a separate Excel file. Each line for each reference was annotated based on the endpoints reported in the monograph text. Examples of endpoints vary widely and depend on the language used directly within the monograph text, ranging from ‘reduced tumour surveillance’ to ‘induce apoptosis in murine haematopoietic cells *in vitro*’. All available details that were provided based on the experimental system (e.g. species or cell line) and assay used were recorded as well. In the latter example provided, ‘murine haematopoietic cells’ would be recorded for the cell line/type, and *in vitro* as the experimental design.

After the list of annotated references had been compiled and organized by experimental design, we downloaded the full-length text PDF of each reference to validate the information reported in the monograph text. If the assay used to measure a particular endpoint/biomarker was not directly identified/reported within the monograph text, the methods section of the primary publication was used to identify the name of the assay used. Similarly, the experimental test system and other related test system details (e.g. animal species or cell type/cell line) were extracted from the methods sections of each primary publication and validated against what was reported in the monograph text, depending on the level of detail initially provided in the IARC Monograph.

Each endpoint measured using a particular assay was extracted to a unique, separate line within the Excel file. If an assay was applied within multiple experimental systems, multiple tissue types/other biosample types, and cell types/cell lines within the same study, these were all considered to be different endpoints and were thus each included as a separate line. Reference information (PMID, DOI, and first author’s last name followed by the year of publication) from which endpoint data was extracted was included on each of these unique lines in three separate column headers, respectively. The extraction of references and information provided from monograph texts and publications was primarily conducted by one team member. The dataset was reviewed in full by other authors on this publication. Subject-matter experts were consulted on specific questions pertaining to relevance of certain endpoints as they relate to KCs, the output/purpose(s) of certain assays, how they are performed, etc., as needed. Data have been mined according to the column headers provided in Supplementary Table 2.

### Organization and synthesis of data

#### Construction of the assay-endpoint list

The list of assays from the references cited in the mechanistic data chapters of the monographs, Smith et al., 2020 [6], and primary publications mined from herein, as well as additional papers reporting on emerging methodologies, constitute a robust database. We sought to make the database both readily searchable and dynamic, with the potential for future expansion to include additional publications, technological advancements/assay development or refinements, and high-throughput *in vitro* or computational models. It is openly accessible and is intended to be subject to feedback and review by the scientific community to gather additional insights, critiques, suggestions, and additions to enhance the quality, relevance, and impact of this work. For example, publications or references to assays that may be used to substantiate that a chemical agent exhibits a KC, including emerging assays/methods, noted to be absent from the database may be submitted to the e-mail address provided on the KC-Assay database (KCAD) landing page.

#### Curation of a user-friendly, dynamic, and readily searchable database

This interactive tool is available at the following Database URL: https://kcad.cchem.berkeley.edu. The front end was developed using HyperText Markup Language and JavaScript, along with Bootstrap for responsive design and Cascading Style Sheets for custom styling. This enables a user-friendly interface for querying and visualizing the data. The backend of the web application is powered by Django, a python-based web framework that handles routing, request handling, and overall data management. The data are stored in a SQLite database, allowing for efficient storage, retrieval, and querying of the data; it is available at the URL provided above. The database has been exported to a PivotTable view, which is a user-friendly tool in data analysis that allows for the summarization and interactive examination of datasets. By reorganizing and summarizing selected columns and rows of data and displaying them in a customizable format, pivot tables enable users to ask targeted questions about their data and identify areas that require further investigation.

## Results and discussion

The extracted data have been organized according to the column headers in Supplementary Table 2. Having multiple columns in a database is invaluable for efficiently managing and querying datasets. This multilevel structuring allows for highly customizable and targeted queries, enabling users to filter and sort data based on their specific research needs or inquiries. The ability to stratify entries by several columns enhances the database’s usability, making it easier to retrieve information relevant to the user more readily.

The results presented herein provide a case study as to how this database may be used in a decision-making context. All abbreviations are summarized in Supplementary Table 3. A summary of the assays conducted in exposed humans *in vivo* pursuant to each KC is provided in Supplementary Table 4(A–J). This list was readily generated by selecting the *in vivo* experimental system type and restricting the species to ‘Human’ only.

### Assays used to measure endpoints in humans

As set forth in the Preamble to the IARC Monographs [2], the body of mechanistic evidence of a chemical agent’s carcinogenic potential is considered to be strong when demonstrated to be ‘consistent and coherent in exposed humans’, in human primary cells or tissues, or in experimental systems (which may include one or a few studies in human primary cells and tissues). Importantly, when there exist a strong mechanistic body of evidence in exposed humans and sufficient evidence of cancer in experimental animals, this is considered sufficient to drive a Group 1 (‘carcinogenic’) carcinogenicity classification for a (chemical) agent. Strong mechanistic evidence in human cells or tissues (primary or otherwise), when accompanied by a minimum of sufficient evidence of cancer in experimental animals, will drive a Group 2A (‘probably carcinogenic’) classification. As such, studies in exposed humans and in human primary cells or tissues that incorporate endpoints relevant to KCs of carcinogens are emphasized when available [3]. Certain KCs, including KC1, KC2, and KC5, are characterized by endpoints for which there are a wide variety of assays that can be used to measure them in exposed humans. Measuring electrophilicity (KC1), genotoxicity (KC2), and a chemical’s capacity to induce reactive oxidative stress (KC5) is more established in the scientific literature because these endpoints involve direct, quantifiable chemical or cellular effects that can be assessed through well-validated and standardized assays.

#### Measurement of KC1 in exposed humans

Assays used to measure the electrophilicity or the ability of a chemical to be metabolically activated have been categorized into four subgroups via this mapping exercise: DNA adducts, RNA adducts, protein adducts, and as to whether a chemical agent exhibits electrophilic behaviour. There are several ‘common’ methods used to measure DNA adducts, RNA adducts, and protein adducts, such as radioactive labelling and mass spectrometry (MS), which can be applied across a range of experimental designs [9–11], while the electrophilic behaviour of a chemical can be modelled *in silico* [12].

Measurement of DNA adducts, an indicator of electrophilic potential, in human tissues, includes [32]P-Postlabeling techniques [13], DNA adductomics [14, 15], immunohistochemistry, and MS (e.g. isotope dilution-MS, electrospray ionization-MS, and linear ion-trap-MS, among others [9]. MS applications similarly have been used to measure electrophilic chemical-adducts on proteins in the body, such as albumin and haemoglobin [16–20]. These methods have been applied, for example, in the following human tissues: bladder, peripheral blood, brain tissues, lung, oral tissues, and saliva/sputum samples [9, 13, 21–23].

#### Measurement of KC2 in exposed humans

Genotoxicity (KC2) describes the ability of chemicals to cause damage to genetic material and can be measured using various assays that detect DNA damage, mutations, or chromosomal alterations. DNA damage is reversible through cellular repair mechanisms, making it less dependable as a measure of potential cancer risk compared to mutations, which are permanent changes passed down through generations of daughter cells and may play a role in cancer development. Tests that detect gene or chromosomal mutations are therefore considered more indicative of carcinogenic potential.

All three of these larger subgroups of genotoxicity (chromosomal damage, DNA damage, and mutations) can be measured in exposed humans. There are several methods used for measuring chromosome damage, including chromosomal aberration assays, fluorescence *in situ* hybridization (FISH) including OctoChrome FISH, micronucleus assays, nuclear aberration assays, pseudoautosomal region–based detection approaches, and the sister chromatid exchange test. Nuclear aberration assay has been used to measure a chemical’s ability to induce chromosome alterations in the hair follicle [24], while all other methods mainly use blood samples obtained from exposed humans [25, 26].

DNA damage is commonly measured using the comet assay, including both the standard comet protocols, which measure double-strand DNA breaks, or the alkaline elution version, which can measure both single- and double-strand DNA breaks, as well as alkali-labile sites, DNA–DNA crosslinking, DNA–protein crosslinking, and incomplete excision sites [27]. The Comet-FISH assay can also be applied to biosamples of exposed humans to measure both single- and double-strand DNA breaks [28]. This group of assays has been applied for example to blood samples (including the buffy coat), the buccal mucosa, the nasal cavity, conjunctival epithelial cells, lachrymal epithelial cells, placental cells, and/or sperm [25, 28–30]. The nick translation assay, also capable of measuring DNA strand breaks, exploits the ability of certain DNA polymerases to add nucleotides to 3ʹ ends of nicks (breaks) in the DNA, where the nucleotides can be tagged with markers. This assay has been conducted in peripheral blood samples of humans exposed to environmental agents [31] and in other experimental systems [32, 33].

Mutation assays can be applied in exposed humans, including the glycophorin A assay and Pig-a-assay (both conducted in erythrocytes), and a T-cell cloning assay used to measure hypoxanthine-guanine phosphoribosyltransferase mutant frequencies in the blood of carcinogen-exposed workers [34]. A restriction fragment length polymorphism approach has been used to measure K-*ras* mutations in pancreatic tumour samples of humans exposed to chemical carcinogens [35]. Moreover, the advances in next-generation sequencing (e.g. high-throughput, error correction, and single cell) provide a powerful tool for examining genomic mutations in exposed humans [36–39]. These assays represent a spectrum of methodologies ranging from specific locus mutations to genome-wide analysis, essential for understanding the genetic impacts of chemical exposure in human populations.

#### Measurement of KC5 in exposed humans

Oxidative damage to DNA, lipids, and proteins provides critical indicators of oxidative stress in the body, reflecting an imbalance between reactive oxygen species (ROS) production and the body’s detoxifying capabilities. Oxidative damage to DNA can be measured in peripheral blood mononuclear cell samples by using a modified alkaline comet assay (modified with lesion-specific endonucleases) [40–42]. MS is also commonly used to measure levels of 8-hydroxy-2ʹ-deoxyguanosine (8-OHdG), 8-oxoguanine (8-oxoGua), 8-oxo-7,8-dihydroguanosine (8-oxoGuo), and 8-oxo-7,8-dihydro-2ʹ-deoxyguanosine (8-oxodGuo) adducts, formed through oxidative damage to the guanine base in DNA and RNA, in primary blood or urine samples from exposed humans [43–45].

Assays measuring oxidative damage to lipids and proteins measure lipid peroxidation and protein oxidation, respectively. Thiobarbituric acid reactive substance assay, which measures malondialdehyde levels, is a well-established means of measuring lipid peroxidation [46], while protein carbonyl formation and tyrosine nitration are used to measure protein oxidation [47]. Reactive product formation can be directly measured in the blood of exposed humans as well, measuring intracellular (highly) ROS [46] and peroxynitrite/peroxynitrous acid nitrogen dioxide radicals [48]. ROS have been measured in other mammalian species using dihydrorhodamine-123 and flow cytometry techniques and may similarly be applied to human blood samples [49].

Antioxidant enzyme activity [50], including superoxide dismutase, glutathione peroxidase, gamma-glutamyl transpeptidase, etc., also provide means of measuring the induction of oxidative stress in blood samples obtained from exposed humans [47, 51]. Reduced glutathione (GSH), an indicator of nonenzymatic antioxidant activity, can be measured using the 5,5ʹ-dithiobis-(2-nitrobenzoic acid) assay [46] or the nonenzymatic GSH recycling assay [47], while vitamin radical scavenger levels (e.g. alpha-tocopherol (vitamin E) concentration/levels or vitamin C/ascorbic acid concentrations/levels), can also be measured in primary human blood samples using High-Performance Liquid Chromatography (HPLC) methods [47].

### KCs with emerging lists of established assays

In contrast, endpoints like epigenetic changes (KC4), immunosuppression (KC7), and modulation of receptor-mediated effects (KC8) are more complex, involving multifaceted biological systems with indirect and variable responses that are harder to isolate and measure consistently. Through this mapping exercise, the IARC monographs have illuminated those that have been commonly applied in the supporting mechanistic evidence (Supplementary Table 4(G), 4(H), and 4(D), respectively) as reliable means of measuring each respective characteristic in exposed humans. Relative to these KCs, this mapping exercise has highlighted the relatively limited number of assays applicable to KC3, KC6, KC9, and KC10 that can be applied in exposed humans. These are summarized in Supplementary Table 4(C), 4(F), and 4(J), respectively.

#### Measurement of KC4 in exposed humans

The induction of epigenetic changes (KC4), which are modifications to DNA, chromatin, or noncoding RNA levels that affect gene expression without altering the DNA sequence (thus resulting in downstream effects), has also been implicated in carcinogenesis. The key processes responsible for epigenetic regulation are changes in DNA methylation patterns, posttranslational gene regulation by noncoding RNAs, chromatin modification (covalent alteration in core histones), and nucleosome positioning (physical changes). Deregulation of these processes causes aberrant gene function and altered gene expression that may play a critical role in cancer initiation, development, and subsequent progression [52]. Similar to KC7 and KC8, KC4 also presents unique challenges in that these changes are often subtle, reversible, context, and directionality-dependent, (i.e. upregulation vs. downregulation), requiring specialized techniques that are not yet standardized or used in regulatory toxicology.

Through this mapping exercise, several KC4 assays or detection techniques have been identified across these different subgroups, many of which can be applied in exposed humans or in primary human tissues or cells. Methods used to measure DNA methylation in exposed humans, both global and locus-specific, include (but are not limited to) the affinity enrichment technique, bisulfite conversion, Enzyme-Linked Immunosorbent Assay (ELISA), application of endonucleases and methyl-sensitive restriction enzymes, HPLC, the luminometric methylation assay, MS, and colorimetric methylated DNA quantification kits [53–55].

Assays measuring changes in the expression of both small and long noncoding RNAs have also been used as supporting evidence of KC4. These methods range from Reverse Transcription-Polymerase Chain Reaction, northern blotting, hybridization to microarrays, cloning, and sequencing, as well as *in situ* hybridization methods. This includes single-cell miRNA detection by microscopy with *in situ* hybridization to measure small noncoding RNAs [56] and single-molecule RNA *in situ* hybridization (smRNA FISH) to measure long noncoding RNAs, a heterogenous class of mRNA-like transcripts that do not code for proteins [57]. A more recent study employed RNA-seq as a means of identifying long noncoding RNA tissue- and cancer-specific expression profiles in human blood or urine samples [58]. For instance, diagnostic tests have been developed for the detection of PCA3, a prostate-specific long-coding RNA (lncRNA), overexpressed in prostate cancer in urine samples as a biomarker for noninvasive diagnosis of prostate cancer [59, 60]. Additional endpoints and assays that have been employed in exposed humans and have been used to support the classification of a chemical as exhibiting KC4 are summarized in Supplementary Table 4(D).

#### Measurement of KC7 in exposed humans

Measuring immunosuppression (KC7) in the body is multidimensional, as it affects the entire immune system and involves interactions between innate and adaptive immunity. As the biological endpoints of immunosuppression are more ambiguous than those associated with genotoxicity, for example, this mapping exercise has aimed to characterize those that have been referenced in previous monographs to refine this list.

Previously, evidence of alterations in the body’s haematopoietic process has been measured by clinical manifestations of haematological disease or conditions (e.g. aplastic anaemia) [61, 62] or through haematopoietic cell population or progenitor cell colony formation through histopathological examination or colony-forming assays, respectively [63–65]. Manifestations of exaggerated immunoglobulin-E (IgE)-mediated immune response, through self-report or observation of conditions such as allergic contact dermatitis [66] or allergic asthma [67–69], etc., associated with chemical exposure, have also been used to substantiate evidence of immunosuppression. Most commonly, haematological parameters, such as differential leukocyte, peripheral blood cell morphology, and total blood count and differential (e.g. white blood cell count), have been used as indicators of immunosuppression in human blood samples [70, 71], as they provide insight into the state and functionality of the immune system. It is for this same reason that lymphocyte immunotyping is used, by measuring cell surface marker expression through various methods (e.g. ELISA [71, 72] or flow cytometry [73]), as detailed analysis of lymphocyte subsets (e.g. CD4+ and CD8+ T cells) can reveal specific immune deficiencies. Additional endpoints and assays that have been employed in exposed humans and have been used to support the classification of a chemical as exhibiting KC7 are summarized in Supplementary Table 4(G).

#### Measurement of KC8 in exposed humans

Chemicals that modulate receptor-mediated effects, such as by mimicking or interfering with the normal signalling pathways of hormones, binding to their receptors, and altering physiological processes, exhibit KC8. This can lead to an inappropriate activation or inhibition of these pathways, promoting abnormal cell growth and division. Endocrine disruptors, specifically, can interfere with the hormonal balance within the body, leading to altered metabolic, developmental, and reproductive processes that can set the stage for cancer. By disrupting hormone function, these carcinogens can change the expression of genes that regulate the cell cycle, potentially leading to uncontrolled proliferation, DNA damage, and the formation of tumours.

This mapping exercise has aimed to characterize endpoints and associated assays used to measure a chemical’s association with KC8. In exposed humans, blood samples have previously been used to measure peptide hormone levels (e.g. human growth hormone, luteinizing hormone, and prolactin) [74, 75], circulating steroid hormone levels (e.g. testosterone) [75], and circulating thyroid hormones [thyroxine (T4) and triiodothyronine (T3)], both free and total [76, 77]. Previously, hyperthyroidism or hypothyroidism as reported by a questionnaire has also been used in epidemiological studies to indicate altered levels of thyroid hormones in human subjects [78], and thyroid function and morphological changes as measured using ultrasound examination have also been employed [79]. Additional endpoints and assays that have been employed in exposed humans and have been used to support the classification of a chemical as exhibiting KC8 are summarized in Supplementary Table 4(H).

### Leveraging new approach methodologies

There is no single harmonized definition of new approach methodologies (NAMs) (also termed Non-Animal Methods). Generally, NAMs refer to innovative, nontraditional testing strategies that include *in vitro* assays, *in silico* computational models and other alternative methods designed to improve and potentially replace traditional animal testing for safety and efficacy assessments. These methodologies aim to provide more human-relevant data, reduce animal use, and enhance the efficiency and predictive power of safety evaluations in regulatory science [80].

Where methods to measure KC-relevant endpoints in humans are lacking, NAMs may be leveraged to improve the overall body of evidence. Our database provides a resource for identifying NAMs that have previously been linked to the KCs. By integrating and cross-referencing data from existing studies, IARC monographs, and publicly available resources, the database allows users to identify which NAMs have been associated with specific carcinogenic endpoints, such as genotoxicity, modulation of receptor-mediated effects, immortalization, etc. A summary of the assays conducted *in vitro* and *in silico* pursuant to each KC is provided in Supplementary Table 5(A–J). This list was generated by selecting for both experimental system types, with no restrictions applied to the species.

#### *In vitro* assays

*In vitro* assays have been applied across all KCs in IARC monographs 112–131, with notable representation in subsections addressing KC3, KC8, and KC10. For example, altered gene and protein expression of critical cell regulatory and DNA repair genes, a potential measure of DNA repair capacity, was assessed in an adenocarcinomic human alveolar basal epithelial cell line, A549 [81, 82] [Supplementary Table 5(B)]. Human cells, such as the immortalized breast cancer cell line MCF-7, are commonly used in the E-screen assay to measure oestrogen receptor (ER) binding. ER transactivation studies, which are frequently cited as evidence of KC8, employ transgenic cell lines such as Chinese hamster ovary, CHO-K1 [83, 84]; rat leiomyoma, ELT3 [85]; or MCF-7 [86] in luciferase reporter gene or B-galactosidase assays [Supplementary Table 5(H]. For KC10, the growth inhibition assay has been used to measure proliferative or antiproliferative effects on tumorigenic cell lines such as AKR2B mouse embryo fibroblast cells, DLD1, and HCT15 [87–89], and the clonogenic survival assay has been applied in immortalized Rat-1 fibroblasts or Syrian Hamster Embryos [90, 91] [Supplementary Table 5(J)]. While these methodologies are not necessarily ‘new’ approach methodologies, they still provide well-established nonanimal methods that contribute to constructing the mechanistic evidence basis for these KCs.

The Toxicology in the 21st Century (Tox21) multiagency initiative, including the US Environmental Protection Agency (USEPA), has been a cornerstone of NAM development geared at evaluating chemical toxicity using high-throughput screening. Tox21 and the USEPA Toxicity Forecaster (ToxCast™) programme makes *in vitro* medium- and high-throughput screening assay data publicly available for prioritization and hazard characterization of thousands of chemicals. The assays employ a variety of technologies to evaluate the effects of chemical exposure on diverse biological targets, ranging from distinct proteins to more complex cellular processes like mitochondrial toxicity, nuclear receptor signalling, immune responses, and developmental toxicity. Importantly, a separate effort to map Tox21/ToxCast™ assays to the KCs of carcinogens is currently underway.

#### *In silico* assays

Computational models that predict the KCs of carcinogens and other toxicities are essential for advancing regulatory science. Tice *et al*. [92] evaluated the availability of *in silico* models for each KC of carcinogens, identifying significant data gaps that hinder the development of comprehensive carcinogenicity protocols for regulatory use. While computational methods exist for assessing KC1 and KC2 as demonstrated in [93] and [94], respectively, models for the remaining KCs still require development. Specifically, *in silico* modelling for KC8 has been referenced in the IARC monographs, with a molecular docking study used to model ER interactions [95].

Additionally, there are important publicly available webservers such as NR-ToxPred [96], which predict agonist and antagonist activity against more than 10 nuclear receptors. For KC8, Borrel and Rudel have developed a cheminformatics approach to identify structural features associated with oestrogen or progesterone steroid production [97]. This tool can be used to help recognize areas where NAMs are well-established and effective, while also highlighting gaps needing further development or validation. This comprehensive approach facilitates targeted research and the continuous improvement of the toxicological evaluation database, ensuring that it remains a dynamic resource for the scientific community.

### Mapping overlapping endpoints can facilitate the assembly of proposed modes of action

Carcinogenesis is a dynamic, multistep, and nonlinear process. The KCs of carcinogens encompass various biological activities and mechanisms that overlap with one another [98]. While KCs are useful in identifying how carcinogens interact with biological systems to cause cancer, KCs are not themselves mechanisms or modes of action (MOAs). For instance, oxidative stress (KC5), genotoxicity (KC2), and chronic inflammation (KC6) are inter-related endpoints frequently observed in carcinogenic pathways. Oxidative stress generates ROS that cause direct DNA damage which may lead to mutations [99]. Oxidative DNA damage can also initiate a chronic inflammatory response in the body, further producing ROS and perpetuating a cycle of damage and repair that contributes to carcinogenesis. Furthermore, inflammation itself releases cytokines and other mediators that can induce additional oxidative stress and DNA damage, demonstrating how these endpoints are interconnected in the development of cancer [100, 101]. These overlapping mechanisms highlight the multifaceted nature of carcinogenesis and the importance of addressing multiple endpoints in cancer hazard evaluation [102]. Researchers can map these assays to multiple KCs, supporting a more holistic evaluation of potential carcinogens and providing an opportunity for an unbiased way of building MOA proposals without *a priori* assumptions [103].

### Database limitations

Manually mining endpoints that can be mapped to adverse events in carcinogenesis pathways presents several ontology challenges. The complexity of biological data, with numerous genes, proteins, and pathways interacting in intricate ways, makes accurate mapping difficult. For some ontologies and pathway ontologies, it is possible to group certain biomarkers within a xenobiotic grouping; however, this grouping might vary depending on the toxicological endpoint being studied. Annotations for these groups might need to create customized target families that are specifically relevant to a carcinogenesis perspective. This ensures that the annotations accurately reflect the biological context and the specific toxicological concerns being addressed.

Additionally, data heterogeneity from various sources like clinical trials, epidemiological studies, and toxicology studies, each using different terminologies, complicates integration into a unified ontology. Standardizing these terminologies, ensuring data quality and completeness, and keeping the ontology updated with evolving scientific knowledge present a time-intensive and challenging task.

Other challenges include scalability and interdisciplinary collaboration, as effective ontology mapping benefits from integration with existing ontologies. These challenges necessitate a combination of robust data management, interdisciplinary expertise, and ongoing updates to maintain accuracy and relevance in the rapidly advancing field of carcinogenesis research.

## Opportunities and conclusions

Our comprehensive database, which was compiled systematically using data from Monographs 112–131 as well as the publication, among other sources, is a useful tool for searching for assays and endpoints relevant to the 10 KCs of carcinogens for chemicals/agents of interest to researchers and regulatory scientists. This database aims to provide context to and guidance regarding the endpoints that assays are measuring, the types of experimental systems they may be applied in, and how they have been previously used to substantiate hazard assessment efforts.

The database is designed as a living document or online toolbox, with the potential for future updates. Advances in text mining technologies, semantic ontology alignment, and improved visualization of association networks can be used to significantly enhance the usability and interoperability of the KCAD. For example, as Natural Language Processing evolves, this technology could be used to automate much of the text mining efforts required to extract relevant assays, biomarkers, endpoints, and other study information that embodies the KCAD from newly published toxicology studies or other hazard assessment reports. For example, data from newer IARC Monographs published since the inception of this project, as well as future Monographs, could be incorporated into the database. This technology could further be used to support integration of other publications detailing emerging assays or methods. Rapid integration of data, especially where the scope of ‘exposure’ type to be expanded beyond chemical agents, as was done for this pilot project, may influence the trends that we have seen thus far in this database. For example, there is a relative sparsity of assays related to KC9, ‘causes immortalization’, as this is a characteristic commonly observed in viruses [4, 104]. Inclusion of lifestyle factors or occupation-related exposures (i.e. night shift work, occupational exposure as firefighter, consumption of red meat, etc.) may also improve the breadth of assays for certain KCs.

In overcoming data heterogeneity (Database limitations section), semantic ontology alignment can harmonize the variations in the terminology used in the toxicology/environmental health literature. Consistency in how assays and endpoints are classified makes data more interoperable and ensures that new scientific literature and diverse datasets can be easily integrated into the KCAD and queried (e.g. matching new data with existing KC or endpoint classifications). Future efforts in aligning KC definitions with existing toxicology and regulatory ontologies (e.g. MeSH [105], the EBI Ontology Look Up Service [106], and the Gene Ontology Consortium [107]) could also improve data integration, allowing researchers to better search for, compare, and interpret mechanistic evidence across multiple datasets. Additionally, cross-mapping terms generated by human experts, as described by [108], can further enhance interoperability by aligning chemical risk assessment terminology with systematic review methodologies, reducing inconsistencies and improving the accuracy of mechanistic data searches [108].

The KCs provide a framework for conceptualizing how the underlying KC-associated endpoints relate to and are linked to one another, which can strengthen the biological plausibility of a chemical exposure and cancer [104]. Interactive network visualization of these associations may be useful in mapping and exploring the dynamic (and often overlapping) relationships between assays, endpoints, and the KCs. Feedback, comments, or other suggestions from the scientific community are welcomed to improve breadth and usability of the current database or for future applications thereof.

## Supplementary Material

baaf026_Supp

## Data Availability

The data underlying this article are available in the Key Characteristics Assay Database at the URL https://kcad.cchem.berkeley.edu, and can be accessed by selecting the download button on the website homepage. Alternately, the data underlying this article can be shared on reasonable request to the corresponding author.
